# Mechanical and elemental characterization of ant mandibles: consequences for bite mechanics

**DOI:** 10.1098/rsfs.2023.0056

**Published:** 2024-04-12

**Authors:** Cristian L. Klunk, Michael Heethoff, Jörg U. Hammel, Stanislav N. Gorb, Wencke Krings

**Affiliations:** ^1^ Animal Evolutionary Ecology, Technische Universität Darmstadt, Schnittspahnstr. 3, Darmstadt 64287, Germany; ^2^ Institute of Materials Physics, Helmholtz-Zentrum Hereon, Geesthacht, Germany; ^3^ Department of Functional Morphology and Biomechanics, Zoological Institute, Christian-Albrechts-Universität zu Kiel, Am Botanischen Garten 1-9, Kiel 24118, Germany; ^4^ Department of Cariology, Endodontology and Periodontology, Universität Leipzig, Liebigstraße 12, Leipzig, Germany; ^5^ Department of Electron Microscopy, Institute of Cell and Systems Biology of Animals, Universität Hamburg, Martin-Luther-King-Platz 3, Hamburg 20146, Germany

**Keywords:** finite-element analysis, functional morphology, hardness, insect, material properties, Young's modulus

## Abstract

Mandible morphology has an essential role in biting performance, but the mandible cuticle can have regional differences in its mechanical properties. The effects of such a heterogeneous distribution of cuticle material properties in the mandible responses to biting loading are still poorly explored in chewing insects. Here, we tested the mechanical properties of mandibles of the ant species *Formica cunicularia* by nanoindentation and investigated the effects of the cuticular variation in Young's modulus (E) under bite loading with finite-element analysis (FEA). The masticatory margin of the mandible, which interacts with the food, was the hardest and stiffest region. To unravel the origins of the mechanical property gradients, we characterized the elemental composition by energy-dispersive X-ray spectroscopy. The masticatory margin possessed high proportions of Cu and Zn. When incorporated into the FEA, variation in E effectively changed mandible stress patterns, leading to a relatively higher concentration of stresses in the stiffer mandibular regions and leaving the softer mandible blade with relatively lower stress. Our results demonstrated the relevance of cuticle E heterogeneity in mandibles under bite loading, suggesting that the accumulation of transition metals such as Cu and Zn has a relevant correlation with the mechanical characteristics in *F*. *cunicularia* mandibles.

## 1. Introduction

Many insects evolved the ability to capture prey and chew food items using their mandibles [1,2]. However, different species also employ their mandibles to perform other tasks besides food processing, like intraspecific contests, defence, transportation, construction and excavation. This multi-task employment of the mandibles raises questions about the role of morphology in functional performance. An approach widely employed to investigate form–function relationships in biological structures is finite-element analysis (FEA). It consists of a numerical method that approximates a structure's mechanical responses to external loads, revealing patterns of structure deformation, strain and stress [3]. A digital representation of the target structure, knowledge about its material properties, and information on its boundary conditions (regions of loading and contact with other structures) are necessary to define a FEA [3].

Regarding insect mandibles, for example, FEA was employed to investigate their mechanical behaviour in Odonata [4], beetles [5], and ants [6–10] considering different employments. However, in those cases, the mandible cuticle was modelled as a homogeneous material, even though it is recognized that the mechanical properties of insect cuticles can vary substantially along the same structure [11–18], with relevant biomechanical effects [11,12,15,19–23]. In a FEA case study on mollusc teeth, which were either homogeneous or heterogeneous in material properties, the effect of the mechanical property distribution on teeth stress and strain patterns could be documented [24]. It showed that when the material was heterogeneous, stresses increased and strains decreased, whereas teeth of the homogeneous material experienced less stress levels and higher strains.

In comparative studies of animal biomechanics using FEA, it is usual to assume that the structure material properties are homogeneously distributed [25] and that their interspecific variation is negligible or is only not the focus of the study. This assumption is not a problem only when the intention is to perform a comparative analysis on objects varying little in their material properties and to investigate the sole effects of morphological variation [3]. However, an ideal approach in organismal biomechanics and functional ecology should explore the role of material property variation, which would also aid in our understanding of the interplay between materials and morphology and provide some insight into the material properties' evolution [15,19–26]. However, researchers usually stumble on the scarcity of data regarding the mechanical properties of biological materials, a consequence of the difficulties associated with such measurements, and the impressive intraspecific and even intraindividual variation that reduces the representativeness of such data at the species level [27].

Two main components of the insect cuticle are the polysaccharide polymer chitin and a series of proteins [28]. One of the most relevant aspects of the cuticle is its sclerotization or tanning, which involves chemical reactions in the exocuticle that change the molecular arrangements between chitin and the protein matrix, modifying the mechanical properties of this cuticular layer, like its stiffness, hardness, strength, among other characteristics [13,29]. Transition metals (Cu, Fe, Mn, Zn) and alkaline earth metals (Ca, Mg) can also bind strongly to the polymers in the chitin increasing the cross-linking density of the fibres and thus the values of the mechanical properties [30–38].

Insect cuticle has essential structural and protective functions, providing the necessary support for muscle anchoring. Also, it protects mechanically and chemically the insect from the environment [39,40]. Several insect behaviours impose relevant mechanical demands on their exoskeleton, like flying, jumping, running, walking, biting and the associated muscle contractions. Such behaviours usually generate friction between body parts and the environment and even among body parts that can lead to cuticular wear, which could also happen with the frequent use of a structure like the mandibles to process hard materials [41–45]. Therefore, it is not surprising that substantial variation in cuticle material sclerotization levels is observed along the body of an insect [15,46–50], besides the differences among the cuticular layers or the abundance of transition or alkaline earth metals. This intraindividual variation in the cuticle can have significant functional relevance due to its effects on the cuticle material properties [15,20–23,51,52].

Among several mechanical parameters, such as the strength, that characterize a material, hardness (H) and Young's modulus (E) provide relevant information on its behaviour under loading [53–56]. Young's modulus is a measure of the material resistance to elastic deformation under compressive or tensile forces, while H reflects the material resistance to localized deformation [57]. These mechanical properties can be related to one another, but do not necessarily have to. Both material properties in biological systems can vary substantially intra- and interspecifically and be modified by the degree of sclerotization or the concentration of specific elements. Elements like Zn, Mn, Ca and Mg were previously found to relate to an increase in cuticle H values and to a high degree of wear resistance of arthropod appendices [11,12,58–67]. Transition and alkaline earth metals in the cuticle are observed in several arthropod lineages, especially on appendages associated with biting or puncture, like spider fangs [12,68], scorpion claws, pedipalps and chelicerae [12,69], mandibles of termites [61], cicadas [70], antlions [50] and ants [11,12,69,71]. Along with these cross-links, it is known that ant species can incorporate minerals into their cuticle [72], similar to the cuticles of crustaceans. All of this highlights the relevance of determining the cuticle elemental composition, as is being explored in the chitinous structures of several mollusc lineages [26,73–75], especially regarding structures heavily employed to perform multiple tasks, like ant mandibles [7,76,77].

Ants show a relevant interspecific variation in mandible morphology [78], whereas intraspecific distinctions are also observed, mainly between worker types [79]. How morphological disparity reflects ant biting performance was investigated for some ant lineages by the use of FEA [6–10] as well as through the estimation of relevant mechanical characteristics based on morphological information [80,81], providing compelling evidence about how mandibular morphological variation can influence bite mechanics, but there has been no attempt so far to simulate the effects of variation in the mandible cuticle mechanical properties in its responses to bite loading.

The present study aims to provide a mechanical and elemental characterization of *Formica cunicularia* Latreille, 1798, worker mandibles, and test through FEA how the mandibular variation in cuticle E influences its responses to bite loading. Based on the distribution of stress observed in ant mandibles in previous attempts to simulate biting behaviour with FEA [6–10], we divided the mandible into four regions, namely the masticatory margin, dorsal (DMA) and ventral (VMA) articulations with the head, and the mandible blade. We hypothesize that the masticatory margin will show the highest value of E and H, followed by the mandibular articulations with the head and the mandible blade. Also, we predict this ranking regarding the proportion of transition and alkaline earth metals along the mandible cuticle. When considering the measured E values in FEA simulations, we expect that there will be differences in relative stress distribution on the mandibles of *F*. *cunicularia* for the biting scenarios tested, comparing simulations with a heterogeneous versus a homogeneous E distribution, in terms of both normalized and non-normalized stress values.

## 2. Results

### 2.1. Mandible exocuticle elemental composition

**Figure 1.**
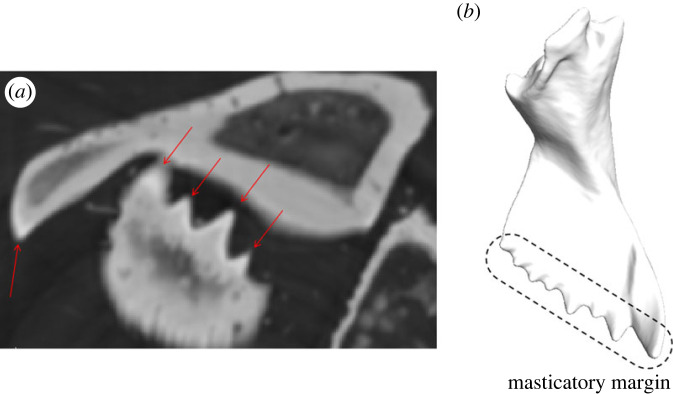
SRµCT scan slice showing the left and right mandibles of a *F*. *cunicularia* worker highlighting the brighter contour of the masticatory margin (red arrows), which suggests the deposition of materials that increase the density of that region (*a*), along with a three-dimensional model of a *F*. *cunicularia* worker mandible showing the general structure of the mandible and specially the masticatory margin (*b*).

The synchrotron radiation X-ray tomography (SRµCT) scans of *F. cunicularia* mandibles showed a distinct white band in the mandible cutting edge (figure 1), suggesting the presence of heavier elements as metals that increase the material density in this region.

**Figure 2.**
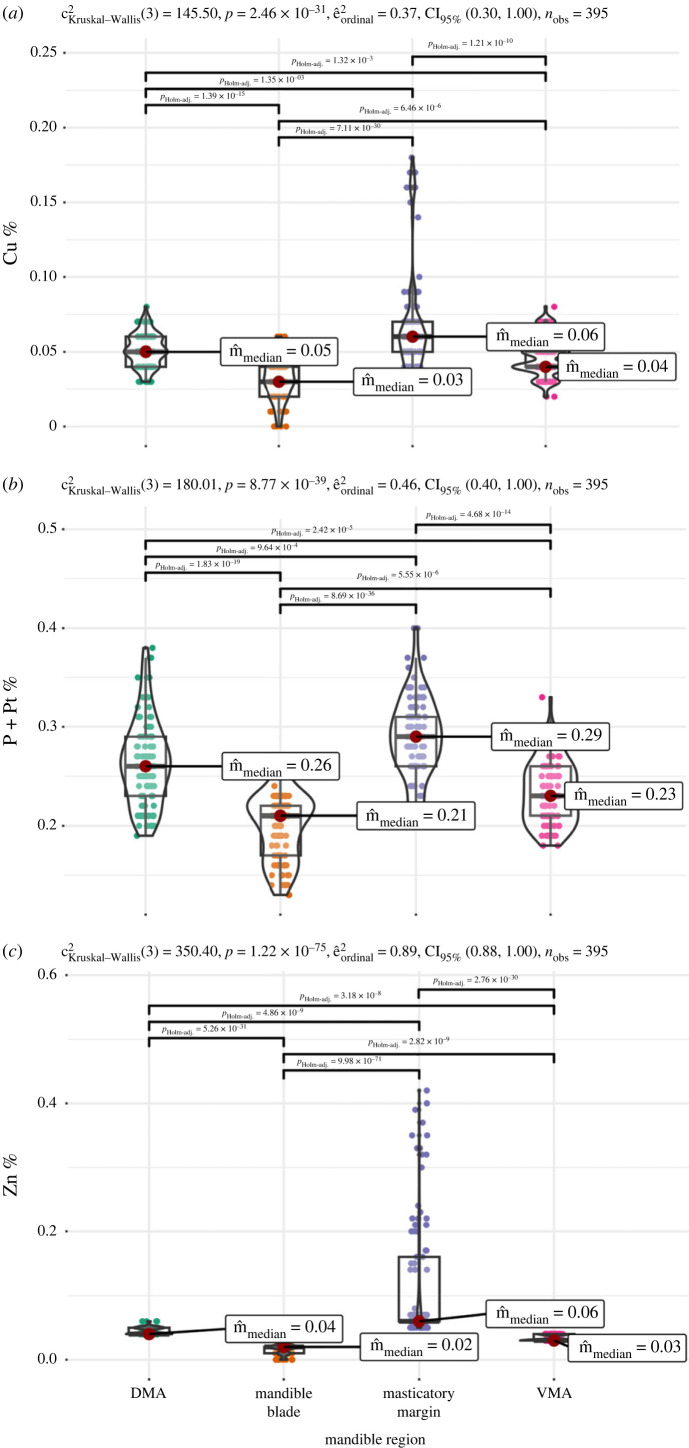
Boxplots depicting the variation in the atomic percentage of Cu (*a*), P + Pt (*b*) and Zn (*c*) in distinct regions of *F*. *cunicularia* worker mandibles, measured with EDS. In the upper left of each graph are depicted the results of Kruskal–Wallis tests for the group difference in atomic % between the mandibular regions, and the horizontal bars connect pairs of mandibular regions that differed in atomic % according to pair-wise Dunn tests (adjusted *p*-values are shown above the bars). Only significant differences are shown. DMA, dorsal mandibular articulation; VMA, ventral mandibular articulation.

According to the energy-dispersive X-ray spectroscopy (EDS) results, some atomic elements occurred in a higher proportion in specific mandibular regions. Notably, Zn, Cu and P + Pt (P and Pt are discussed together because the peak of P overlaps with one of Pt) were found at higher proportions in the mandibular masticatory margin, followed by DMA and VMA, exhibiting lower levels in the mandible blade (figure 2). Some elements showed differential concentrations at specific mandibular regions (i.e. Cl, F, Fe, K, Mn and Si; electronic supplementary material, figure S2), while others were present at similar levels along the entire mandible (i.e. Ca, Mg, Na and S; electronic supplementary material, figure S2).

### 2.2. Mechanical properties

**Figure 3.**
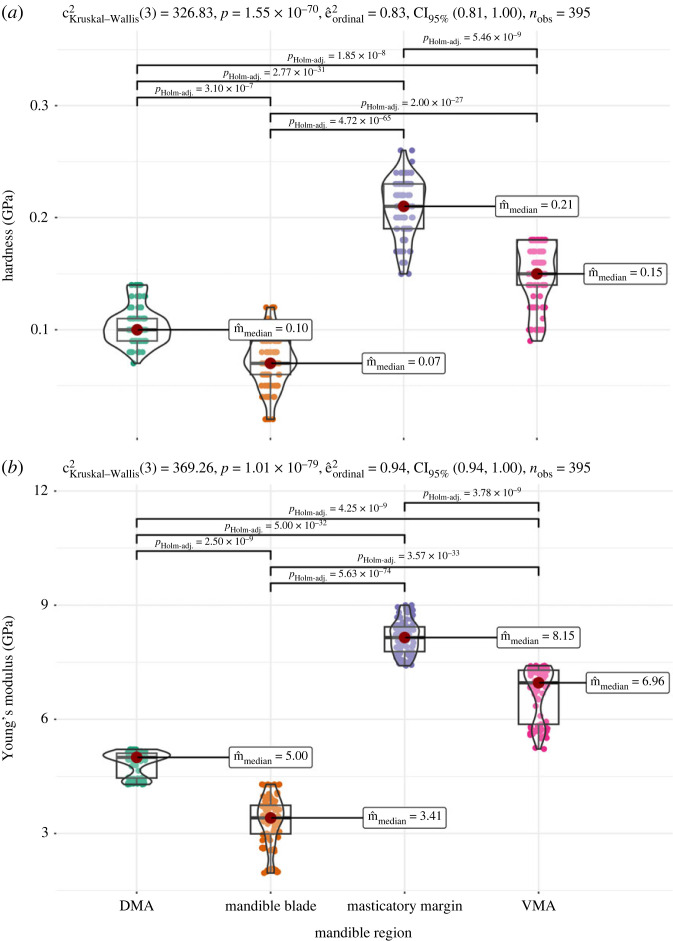
Boxplots depicting the variation in exocuticular H (*a*) and E (*b*) in distinct mandibular regions of *F*. *cunicularia* workers, measured with nanoindentation. In the upper left of each graph are depicted the results of Kruskal–Wallis tests for the group difference between the mandibular regions, and the horizontal bars connect pairs of mandibular regions that differed in the respective mechanical properties according to pair-wise Dunn tests (adjusted *p*-values are shown above the bars). Only significant differences are shown. DMA, dorsal mandibular articulation; VMA, ventral mandibular articulation.

Values of E and H were positively correlated (Spearman's *ρ* = 0.92). Nanoindentation tests showed that the masticatory margin of *F*. *cunicularia* worker mandibles has higher values of exocuticular H and E (figure 3), which agrees with the pattern of cuticle density contrast depicted in the SRµCT scans (figure 1). The mandibular articulations with the head also exhibited higher exocuticular H and E, with the VMA showing higher values than the DMA of both material properties (figure 3). Finally, the mandibular blade presented the lowest levels of exocuticular H and E (figure 3).

### 2.3. Relationship between variables

**Figure 4.**
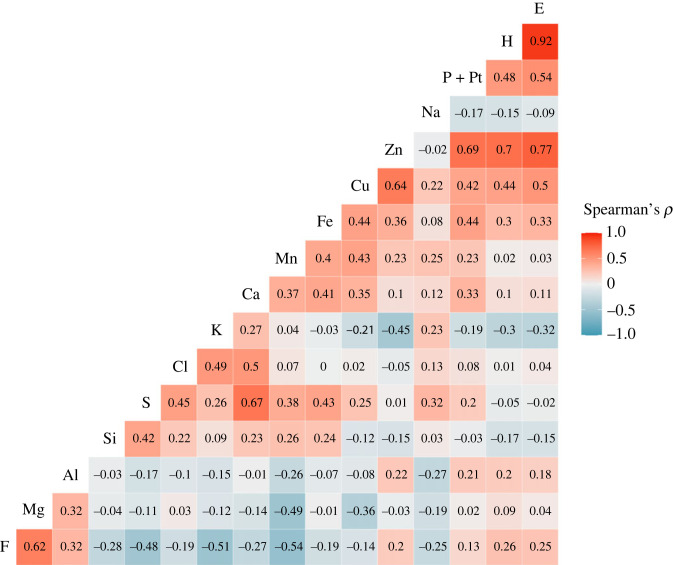
Spearman's rank correlations among exocuticular material properties (H and E) and the proportion of elements for both mandibles of three *F*. *cunicularia* workers. Bluish and reddish colours represent negative and positive correlations, respectively.

We found relevant correlations between the proportion of some atomic elements and cuticle material properties. For exocuticular H, Zn showed the highest correlation (Spearman's *ρ* = 0.70; figure 4). Regarding E, Zn (*ρ* = 0.77), P + Pt (*ρ* = 0.54) and Cu (*ρ* = 0.50) showed the highest correlation values (figure 4). There were also relevant correlations between atomic elements. The presence of Zn was correlated with P + Pt (*ρ* = 0.69) and Cu (*ρ* = 0.64; figure 4). The occurrence of F correlates negatively with the presence of K (*ρ* = −0.51) and Mn (*ρ* = −0.54) and positively with Mg (*ρ* = 0.62; figure 4). The distribution of K was correlated with Cl (*ρ* = 0.50; figure 4). Finally, the occurrence of S and Ca was also correlated (*ρ* = 0.67; figure 4).

### 2.4. Finite-element analysis

**Figure 5.**
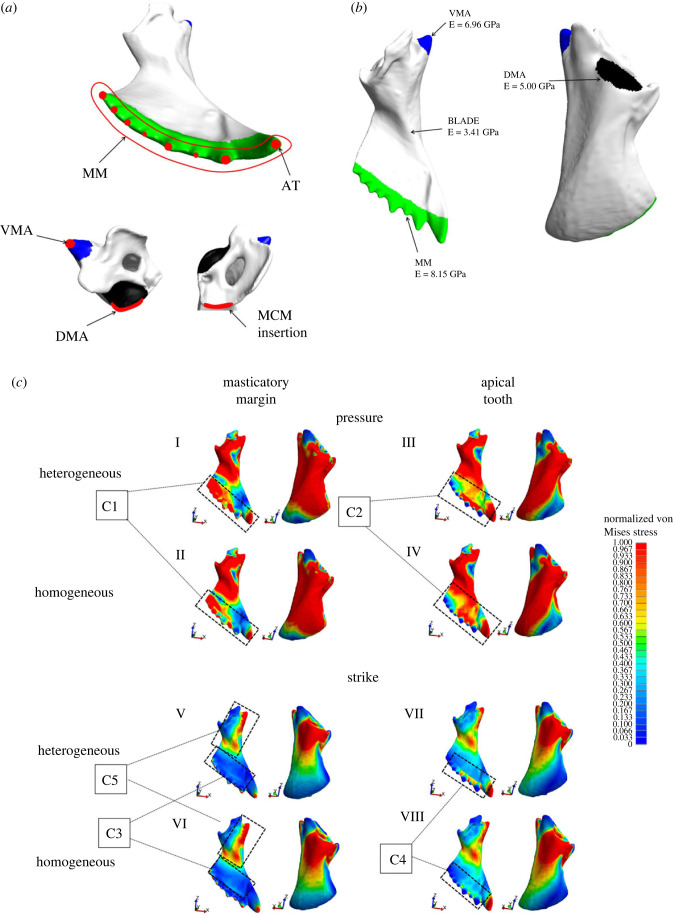
(*a*) Schematic of FEA boundary conditions. Highlighted in red are the mandibular regions where nodal forces and nodal displacement restrictions were defined for biting simulation, as detailed in the main text; AT, apical tooth; BLADE, mandibular blade; DMA, dorsal mandibular articulation; MCM, mandibular closing muscle; MM, masticatory margin; VMA, ventral mandibular articulation. (*b*) Mandibular regions and respective E values as defined for FEA. For the homogeneous treatment, we employed the E value of the mandibular blade to the entire mandible. (*c*) Colour maps depicting von Mises stress distribution along the mandibles for all simulations. Stress values were normalized to allow for direct comparisons between simulations. Therefore, the colour key indicates proportional stress values based on each simulation's defined higher stress value. Pressure with the entire masticatory margin (I–II) or the apical tooth only (III–IV) under heterogeneous (I and III) or homogeneous (II and IV) E; strike with the entire masticatory margin (V–VI) or the apical tooth only (VII–VIII) under heterogeneous (V and VII) or homogeneous (VI and VIII) E. C1–C5: comparisons between E treatments in the corresponding bite simulations, as explored in the main text.

The FEA colour maps depict subtle but consistent differences in stress patterns between simulations with homogeneous and heterogeneous E (figure 5*c*). Most biting behaviours showed a relatively higher stress concentration on the masticatory margin and relatively lower stresses on the surrounding mandible blade under a heterogeneous E treatment (figure 5*c*; C2, C3, C4). The only exception regards strike biting with the entire masticatory margin (figure 5*c*(I–II)), where stresses at the masticatory margin were relatively higher when a heterogeneous E was modelled, but it did not result in a relative decrease of stress levels in the adjacent mandible blade, in comparison with the homogeneous E treatment (figure 5*c*; C1). Similarly, relative stresses in the mandible blade around VMA decreased when the mandible was modelled with a heterogeneous E under a strike biting with the entire masticatory margin (figure 5*c*; C5). No evident differences in relative stress distribution between E treatments were found around the DMA (figure 5*c*).

**Figure 6.**
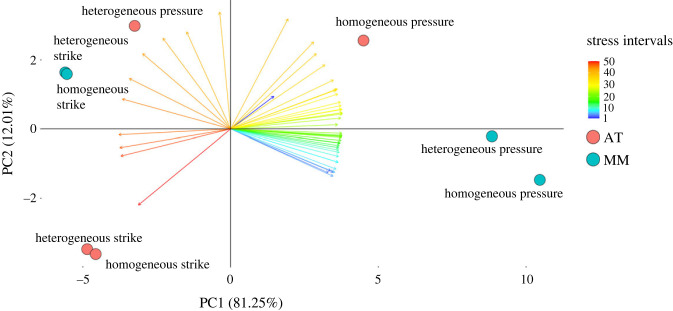
PCA regarding the intervals method depicting the distribution of biting simulations based on the proportion of mandibular volume filled by distinct von Mises stress intervals. Coloured circles represent simulations using the apical tooth only (red; AT) or the entire masticatory margin (blue; MM). Coloured arrows depict distinct stress intervals (from 1 to 50).

To estimate how the *F*. *cunicularia* mandible is influenced by the heterogeneous distribution of E in terms of the amount of mandibular volume filled by distinct ranges of non-normalized stress values, we generated a principal component analysis (PCA) following the intervals method. We expected that for each biting behaviour, the simulations with different E treatments would be distant from each other in the multivariate space. When considering the distribution of stress intervals, the first two components of the PCA explained more than 93% of the variance. PC1 was associated with a range of stress intervals from moderate low towards higher stress intervals, while PC2 showed a stronger association with intervals 39–43 (figure 6). The effects of modelling the mandible cuticle as a homogeneous or heterogeneous material were more relevant under pressure biting, especially regarding the use of the apical tooth only (figure 6). In this case, the most relevant stress interval to separate the E treatments was the highest stress interval, which filled a broad mandibular volume in pressure biting with the apical tooth under a heterogeneous E treatment (interval 50; electronic supplementary material, file S3). Irrespective of E treatment, pressure biting was more associated with the lowest stress intervals, and strike biting was more related to the highest stress intervals, mainly when only the apical tooth was employed for biting (figure 6).

Differences in stress patterns were driven by differential stress concentration at each mandibular region between the E treatments. Specifically, there were relevant differences in median stress values in the mandible regions when comparing simulations with homogeneous or heterogeneous E. In strike biting with the masticatory margin, the DMA (*W*_Mann–Whitney_ = 1.41 × 10^+08^, *p* < 0.001), VMA (*W*_Mann–Whitney_ = 7.61 × 10^+07^, *p* < 0.001) and the masticatory margin (*W*_Mann–Whitney_ = 7.42 × 10^+08^, *p* < 0.001) showed higher median stress values, while the mandibular blade exhibited lower median stress values (*W*_Mann–Whitney_ = 3.04 × 10^+10^, *p* < 0.001) (electronic supplementary material, figure S3). A similar pattern was observed in strike biting with the apical tooth only (electronic supplementary material, figure S5). Regarding pressure bite with the entire masticatory margin, the mandible blade again showed lower stress values under a heterogeneous E distribution in comparison to a homogeneous distribution (*W*_Mann–Whitney_ = 3.00 × 10^+10^, *p* < 0.001), while the masticatory margin (*W*_Mann–Whitney_ = 7.68 × 10^+08^, *p* < 0.001), VMA (*W*_Mann–Whitney_ = 7.17 × 10^+07^, *p* < 0.001), and DMA (*W*_Mann–Whitney_ = 1.77 × 10^+08^, *p* < 0.001) exhibited higher stress values (electronic supplementary material, figure S4). The same general pattern was observed in pressure biting with the apical tooth only, although no difference in stress values was observed regarding the VMA (electronic supplementary material, figure S6).

## 3. Discussion

Mechanical properties strongly affect solid structures. The stiffness E is a measure of a material's capacity to resist deformation, which means that a stiffer material shows a lower degree of deformation than a flexible one under load. The hardness H is a measure of the resistance to localized deformation or indentation, which means that hard materials are more difficult to scratch and can resist any shape change in local areas. The strength is the ability of a material to resist fracture under load. Even though these three material properties describe different behaviours, there seem to be direct relationships between them. Hardness and strength can be correlated as hard materials can be difficult to break and can withstand high stresses. Young's modulus and H can be also correlated, which accordingly is the case in the mandibles of *F. cunicularia*.

Our elemental and mechanical characterization of *F*. *cunicularia* worker mandibles demonstrated that distinct mandibular regions have varying elemental and mechanical attributes, reflected in mandible responses to bite loading. The mandibular masticatory margin showed, in general, a higher material density than the remaining mandible. This mandibular region was stiffer and harder than the remaining mandible and showed a higher concentration of Zn, Cu and P + Pt. When the measured heterogeneity in cuticular E was considered on bite simulations, this region tended to concentrate more stress that otherwise would spread towards the mandibular blade, similar to snail teeth [24]. Due to the high values of H, the masticatory margin is probably less affected from failure and consequently allows for a higher stress concentration in this area. This could be tested in the future in the form of fracture or breaking stress experiments. Potentially, the high concentrations of Cu and Zn primarily increase the hardness, whereas the stiffness is increased as a side effect. This can, however, not be tested.

Interestingly, the mandibular articulations with the head also showed relevant distinctions compared to the mandible blade and masticatory margin. Both DMA and VMA were stiffer and harder and possessed higher levels of Zn, Cu and P + Pt than the mandible blade, showing lower levels of all those attributes than the masticatory margin. The mandible blade showed the lowest levels of exocuticular H and E and concentrated a lower proportion of Zn, Cu, and P + Pt than the rest of the mandible. In biting simulations under a heterogeneous E distribution, the stiffer mandibular regions (masticatory margin, VMA, and DMA) tended to concentrate relatively higher stress levels. This pattern was associated with a decrease in the amount of stress spreading towards the mandible blade, which usually has a thinner and less stiff cuticle, resulting in stress patterns that potentially optimize the biting capacity of *F*. *cunicularia* workers.

A higher degree of exocuticular H and E along the mandible masticatory margin was observed before in ants [11,12,14], although only a few species have been investigated so far, mainly leaf-cutter ants. Such higher values of material properties along the masticatory margin usually follow the concentration of transition metals like Zn [12], which tend to accumulate along the mandible cutting edge or other cutting and piercing tools of arthropods [12,50,70,71]. In leaf-cutter ants, the mandible cutting edge tends to be stiffer and harder [11,12], which could be a mechanism to deal with the abrasion associated with the behaviour of cutting leaves, as well as to improve the self-sharpening mechanism of such a structure [82]. Worker mandibles wear off during their lifetimes, and older workers with less sharp mandibles tend to change their task rules in the colony to focus on carrying leaves or other activities not related to cutting leaves [41] due to the increased amount of forces needed to cut leaves employing worn mandibles [45].

Distinct from the specialized leaf-cutter ants, *F*. *cunicularia* has a morphologically monomorphic worker caste, whose foragers exhibit a generalist behaviour [83]. Also, this species usually ranks at a low position in competitive hierarchies, avoiding physical conflict with other ant species [84]. Our results demonstrate that even one generalist and non-aggressive ant species invest in transition metal accumulation in their worker mandibles, leading to a heterogeneous distribution of material properties, suggesting that such patterns may be widely present in other ant lineages. The potential relevance of a stiffer and harder masticatory margin and mandibular articulations for the biting behaviour and division of labour needs to be further studied for *F*. *cunicularia* colonies. Our results suggest that, at least for the masticatory margin, the effects regarding mandible integrity could be similar to what is observed for leaf-cutter ants [11,12].

The evolution of a dicondylic mandibular articulation with the head occurred very anciently in insects and is prevalent in current lineages [2,85], albeit some reversals to the ancestral condition with a unique point of articulation were suggested [86]. A dicondylic joint reduces the mandibular movement to a single rotation axis but provides an increased stabilization relevant to generating stronger bites [85,87]. By being constrained, joint regions generate reaction forces that impose substantial mechanical demands on the associated structures. Although demonstrated that such reaction forces are relevant for insects during bite [4], there has been no attempt so far to characterize the ant mandible cuticle considering the possibility of the mandibular joints being differentially stiffer than other mandibular regions. Accordingly, the functional relevance of modelling material heterogeneity was demonstrated for some organisms and structures [15,19–24] but rarely regarding bite mechanics. Our results suggest that stiffer regions of the mandible, like the masticatory margin and mandibular joints, can concentrate relatively higher stress levels, reducing the stresses that achieve the thinner mandibular blade. These stress patterns based on the specific distribution of material properties are probably relevant to fulfilling the function of cutting and chewing when high stresses are required at the masticatory margin. At the same time, the surrounding cuticle with lower E and H values could serve as a shock absorber, as more flexible regions allow deformation and could thus prevent the accumulation of high stress when loaded, potentially due to a reduced local bending of the material.

Differences in stress patterns between heterogeneous and homogeneous E treatments under bite loading were subtle, demanding a range of FEA results that included colour maps, distribution of stress intervals, and differences in median stress levels among the mandibular regions to be evaluated. However, even little differences in stress concentrations might be decisive for the structure's proper functioning and simultaneous damage risk minimization. The fact that we were able to demonstrate that the material heterogeneity of the *F*. *cunicularia* worker mandible has functional relevance suggests that such an approach should be applied to other ant species, notably specialized ants in terms of biting demands, like leaf-cutter and trap-jaw ants, whose bite mechanics has been poorly explored besides the relevance of the mandible morphological variation so far [6,8–10,80,81] (but see [11,12,14]).

## 4. Conclusion

Our results draw attention to the necessity to widely explore the variation in cuticular elemental and mechanical characteristics of chewing insect mandibles, especially to investigate their functional relevance under bite loading. Ant workers use their mandibles to perform roughly all non-reproductive tasks they are responsible for, characterizing the multitasking aspect of this working tool. Although the role of mandibular morphological variation proves to be relevant to bite mechanics, we still know little about how the mandible cuticle microstructure can influence its mechanical responses to task-related loading demands.

## 5. Methods

### 5.1. Ant specimens

*Formica cunicularia* is a common European ant, especially abundant in Central Europe [88] and frequently found in urbanized areas. Specimens of *F*. *cunicularia* were collected from a colony located in an urban area in Darmstadt, Germany, in 2022 and stored in 70% EtOH. They are now deposited at the Leibniz Institute for the Analysis of Biodiversity Change, Hamburg, Germany. It is recognized that the conditions of sampling storage affect the material property values, mainly due to their effects on the sample's hydration state [16,89], but the analysed specimens were stored under the same conditions, so the chemical or mechanical characteristics are comparable. We recognize that the specific conditions of those samples' storage prevent the direct comparison of material properties’ values with measurements from other efforts available in the literature, but this should not prevent us from testing our hypotheses related to the distinct mandibular regions.

### 5.2. Mandible scans

With appropriate imaging techniques, it is possible to observe gradients of material density that suggest the accumulation of heavier atoms, like heavy metals. Phase contrast imaging methods allow for the detection of heterogeneity in material density, which points to regions with potential differences in elemental composition (deposition of heavy metals) and consequent variation in material properties. Mandibles from one *F*. *cunicularia* worker were scanned using SRµCT at the Imaging Beamline P05 (IBL) [90–92] operated by the Helmholtz-Zentrum-Geesthacht at the storage ring PETRA III (Deutsches Elektronen Synchrotron—DESY, Hamburg, Germany), through a quantitative phase contrast method using structured Talbot array illuminator (DPC). Tomographic reconstruction was done with a custom reconstruction pipeline [93] using MatLab (Math-Works) and the Astra Toolbox [94–96]. To visualize the distribution of cuticle density and to pre-segment the mandible for biomechanical simulations, we imported the scan data to the software Amira 5.4 (Visage Imaging GmbH, Berlin, Germany). Snapshots of mandible cross-sections were taken to illustrate the occurrence of mandibular regions with higher density (whitish voxels). To generate a surface model of the *F*. *cunicularia* worker mandible, we manually segmented the left mandible using the Amira 5.4 Magic Wand tool at intervals of 10 slices. This pre-segmented mandible file was uploaded to the online platform Biomedisa [97] for automatic interpolation between the pre-segmented slices and the generation of the mandible surface representation. Finally, the outputs from Biomedisa were imported back into Amira 5.4 to correct for inaccuracies and reduce the complexity of the reconstructed morphology.

### 5.3. Exocuticle elemental composition

To investigate the elemental composition of the mandible cuticle, we employed EDS on three *F*. *cunicularia* workers (six mandibles). Here, we used the one specimen previously scanned and two additional ones. To test our main hypotheses that mandibular regions vary in their chemical composition and consequently in their mechanical characteristics, we divided each mandible into four regions, namely the masticatory margin, mandible blade, ventral (VMA) and dorsal (DMA) mandibular articulation with the head.

Mandible samples were cleaned in 70% EtOH with an ultrasonic bath for 20 s. Afterwards, they were attached to glass slides with double-sided adhesive carbon tapes and dried at room temperature. Then, each mandible was surrounded by a small metallic ring posteriorly filled with epoxy resin (Reckli Epoxy WST, RECKLI GmbH, Herne, Germany). After polymerization, lasting for 3 days at room temperature, glass slides and tapes were removed. Each sample was subsequently polished with sandpapers of different roughness until the regions of interest in cross-section were on display. Then, the surface was smoothed with aluminium oxide on a polishing machine (Minitech 233/333, PRESI GmbH, Hagen, Germany), and each sample was cleaned by an ultrasonic bath in 70% EtOH, lasting 5 min to remove the polishing powder. After mounting the embedded samples on scanning electron microscope (SEM) sample holders, they were sputter-coated with platinum (5 nm layer). The platinum was necessary as a reference to check if the EDS measurement was correct (e.g. in some cases, no or very high Pt content was detected, and these measurements were excluded from analyses).

Measurements took place at the exocuticle of different mandibular cross sections and were performed with an SEM Zeiss LEO 1525 (One Zeiss Drive, Thornwood, New York, NY, USA) equipped with an Octane Silicon Drift Detector (SDD) (micro analyses system TEAM, EDAX Inc., New Jersey, USA). For each measurement, the same settings were used (i.e. an acceleration voltage of 20 kV, working distance, lens opening, etc.). Before analysis, the detector was calibrated with copper. Overall, 395 small areas (no mapping and no point measurements) were investigated by EDS. The following elements were selected in the software to measure their proportions: H (hydrogen), C (carbon), N (nitrogen), O (oxygen), Pt (platinum), Al (aluminium), Ca (calcium), Cl (chlorine), Cu (copper), Fe (iron), K (potassium), Mg (magnesium), Na (sodium), P (phosphorus), S (sulfur), Si (silicon) and Zn (zinc). Some elements are not discussed here as they are either the elemental basis of chitin and proteins (H, C, N, O), the coating (Pt), or the polishing powder (Al, O). We also performed 10 EDS tests on the epoxy to identify putative pollution due to the mechanical application, embedding, or polishing. We could not detect Si (part of the sandpaper) or any other elements that we further discussed in the resin. Therefore, their presence is considered part of the mandible. The peak of P overlaps with that of Pt. Because of this, the software could not discriminate between these two elements, so P content could not be reliably determined. Therefore, P and Pt are discussed together (P + Pt). We, however, measured 20 areas of pure epoxy to obtain values on their Pt content (mean ± s.d.; 0.16 ± 0.02 atomic %) to further estimate the proportions of P in the cuticle. We only included measurements regarding element peaks higher than background noise. After EDS analyses, samples were employed for nanoindentation.

### 5.4. Material properties

Both cuticular H and E can be measured through nanoindentation [98]. In nanoindentation experiments, the material surface of a structure is indented with an object of specific geometry (here, a Berkovich indenter tip) and a known force, resulting in material deformation. We used a nanoindenter SA2 (MTS Nano Instruments, Oak Ridge, Tennessee, USA) equipped with a dynamic contact module (DCM) head. We employed the continuous stiffness mode to estimate the material H and E, using indentation-generated force–displacement curves from the loading and unloading phases [98]. All tests were conducted under standard room conditions (relative humidity 28–30%, temperature 22–24°C), with each indent and corresponding curve manually controlled. Values of E and H were determined at penetration depths of 600–1000 nm. We obtained approximately 50 values for each site indented, obtained at different indentation depths, which were averaged to provide one H and one E mean value per indent.

Nanoindentation tests were run on cross-sections of the mandible exocuticle from the three *F*. *cunicularia* workers used for EDS analyses (six mandibles). Indentations were applied to the same localities as for EDS analyses, allowing us to relate elemental composition with mechanical properties. After a region of interest was tested by EDS and nanoindentation, the sample was polished and smoothened again until the next target region was on display. In total, 395 sites were tested by EDS and indentation. Nanoindentation and EDS data are available in the electronic supplementary material, file S1.

### 5.5. Finite-element analysis

We generated the mandible volumetric mesh and performed the FEA in the open-source software FEBio [99]. To define mesh density, we ran convergence tests, ending up with a mesh composed of 317 873 ten-node quadratic tetrahedral elements (TET10), 102 824 faces and 525 717 nodes (electronic supplementary material, file S2), employed in all simulations. Localized refinement was performed around the masticatory margin and the mandibular articulations with the head to improve the resolution at the regions of E transition (electronic supplementary material, figure S1). We simulated four biting scenarios, namely strike and pressure with the entire masticatory margin or the apical tooth only (figure 5*a*). A strike bite consists of a fast-mandibular impact against an object, whereas a pressure bite simulates the behaviour of employing the mandibles to crush an object. For strike biting, we applied a nodal force on the tip of the apical tooth or the tips of the remaining teeth representing the masticatory margin, simulating an impact on the teeth tips (figure 5*a*). To approximate the mandible fixation in the worker head, which is composed of two articulation points, we restricted to zero the nodal displacement in *x*, *y* and *z* directions on the VMA and DMA, maintaining the mandible fixed during biting (figure 5*a*). For pressure biting, we applied a nodal force on the region of the mandibular apodeme insertion, which represents the insertion of the mandibular closing muscles and hence its contraction. Again, to define the mandible articulations with the head we restricted to zero nodal displacement in *x*, *y* and *z* directions on the DMA and VMA. Finally, we simulated the use of the masticatory margin or the apical tooth in pressure biting by restricting to zero the nodal displacement in the tips of the masticatory margin teeth or apical tooth in *x*, *y* and *z* directions (figure 5*a*), employing the same nodes where forces were applied in strike biting. In all simulations, we implemented a 100 000 nN load. We considered the measured E to all predefined mandibular regions to compare FEA results against simulations with a homogeneous E distribution, defined by the value of the mandibular blade (figure 5*b*). Regarding Poisson's ratio, we applied a value of 0.3 to all simulations, as commonly considered for FEA with ant specimens [6–10]. We compared FEA results between E treatments through colour maps and stress intervals [100]. Since an object's state of stress is represented by multiple components [101], interpreting stress dissipation along a structure can be complex. To circumvent this issue, we adopted the von Mises stress [102] as an effective stress value for each element of our simulations. Although not necessarily the best descriptor of the cuticle mechanical behaviour, the von Mises stress is widely employed as an effective stress result in FEA of biological structures.

### 5.6. Statistical analyses

We applied the intervals method [100] to compare the distribution of von Mises non-normalized stress values between each biting simulation with a heterogeneous E distribution and its homogeneous counterpart. Specifically, we adopted the proportion of mandibular volume filled by a defined range of stress values as input variables to generate orthogonal axes through a PCA [100]. We extracted data on von Mises stress (for the generation of stress intervals) and volume (to calculate the mandibular volume) for the elements of each simulation from FEBio [99]. Then, we removed elements representing the 2% higher stress values in each simulation, as these values often represent artificially high stress values [100,103]. We log-transformed stress values before generating stress intervals to account for variation in the scale of non-normalized von Mises stress values. We defined the upper threshold value to let the 25% higher stress values above the threshold, representing the highest stress interval. To define the ideal number of stress intervals, we generated datasets with different numbers of intervals (5, 10, 15, 25, 50, 75) and performed PCAs. We considered the PC1 and PC2 scores of each dataset in linear regressions with the scores of equivalent PCs of the next interval (e.g. PC1_5__intervals∼PC1_10__intervals), and we retrieved the coefficient of determination (*R*^2^) to analyse the convergence of PC scores. The stop of increase in *R*^2^ defines the final number of intervals [100]. Convergence occurred with 50 intervals, so we used this number of intervals for the PCAs (electronic supplementary material, file S3). We conducted the PCA with the R [104] packages FactoMineR version 2.4 [105] and factoextra version 1.0.7.999 [106]. With this PCA we aimed to estimate how much each simulation differs in the distribution of non-normalized von Mises stress values. Therefore, the greater the distance between simulations along the PCA axes, the higher their differences in non-normalized stress distribution.

We tested for differences in element atomic proportion and material properties between the distinct mandibular regions with Kruskal–Wallis tests, applying Dunn tests with Bonferroni corrections to assess paired differences between mandibular regions. We also tested for differences in non-normalized stress values of each mandibular region between the E treatments (electronic supplementary material, file S4) with Mann–Whitney tests, excluding the 2% higher stress values of each simulation as performed for the intervals method procedure. To test for correlations among E and H along the mandible samples, and between those mechanical properties with cuticle element composition, we applied Spearman's correlation. Statistical analyses were carried out in the R [104] environment, where we employed the package ggstatsplot [107] to generate violin plots and perform the Kruskal–Wallis, Mann–Whitney and pair-wise comparison tests. R code is available as electronic supplementary material, file S5.

## Data Availability

Data on E, H and elemental proportion measurements (electronic supplementary material, file S1), finite-element mesh (electronic supplementary material, file S2), proportion of mandibular volume filled by each of the 50 stress intervals for all simulations (electronic supplementary material, file S3), element stress and volume data of each simulation (electronic supplementary material, file S4) and R code (electronic supplementary material, file S5) are available as electronic supplementary material [108].
